# Editorial: Endocrine and cardiometabolic complications of obesity in children: possibilities for reversal in short and long-term observation

**DOI:** 10.3389/fendo.2024.1458355

**Published:** 2024-08-12

**Authors:** Vesna Herceg-Čavrak, Julio Alvarez-Pitti, Michal Brzezinski, Malgorzata Wojcik

**Affiliations:** ^1^ Faculty of Health Sciences, Libertas International University Zagreb, Zagreb, Croatia; ^2^ Innovation in Paediatrics and Technologies-iPEDITEC- research group, Fundación de Investigación, Consorcio Hospital General, University of Valencia, Valencia, Spain; ^3^ Department of Pediatrics, Gastroenterology, Allergology and Pediatric Nutrition, Medical University of Gdansk, Gdansk, Poland; ^4^ Department of Pediatric and Adolescent Endocrinology, Pediatric Institute, Jagiellonian University Medical College, Cracow, Poland

**Keywords:** obesity, obesity in adolescence, intervention - behavioral, obesity complications, obesity intervention strategies

Obesity is currently one of the most significant global health challenges (1). In 2021, the European Commission recognized obesity as a chronic relapsing disease that serves as a gateway to various non-communicable diseases, including diabetes, cardiovascular diseases, and cancer (2). According to WHO data from 2016, 41 million children under the age of 5 and 340 million children aged 5 to 19 were overweight or obese (3).

Childhood obesity is not merely a problem of excessive weight gain. It is a complex, multifactorial condition that leads to a range of endocrine and cardiometabolic complications (4, 5). The majority of overweight and obese children become adults with obesity and its associated complications (6, 7).

This Research Topic brings together a collection of studies that highlight the interconnections between obesity and various health problems in children, advocating for a comprehensive, multidisciplinary approach to management and reversal. However, not all mechanisms that lead to obesity-related complications and diseases are fully understood.

One such study by Bugajska et al., published in this Research Topic, shows that an abnormal amino acid profile in overweight and obese prepubertal children, accompanied by elevated ALT and uric acid levels, may indicate early metabolic disturbances that could lead to metabolic syndrome or metabolic dysfunction-associated fatty liver disease (MAFLD).

Biomarkers are also emerging as essential tools for assessing cardiovascular risk. In the study by Mihuta et al., the link between serum trimethylamine N-oxide (TMAO), obesity, and vascular damage in children was confirmed. Children with obesity and high HOMA-IR presented greater weight excess and significantly higher vascular markers, although TMAO levels did not significantly differ from those in obese children with lower HOMA-IR<cut-offs.

Recent research by Stanikova et al. reveals a significant link between thyroid hormones and body weight changes in obese adolescents, emphasizing the potential of thyroid function as a predictive tool for weight management. The study found that adolescents with increased BMI during the follow-up had significantly higher baseline levels of thyroid-stimulating hormone (TSH) and free thyroxine (FT4) compared to those in the BMI decrease group. Adolescents with obesity and higher baseline TSH and FT4 levels are at a higher risk of natural course weight gain/BMI increase. Integrating insights from thyroid function into clinical practices may refine treatment options and enhance outcomes for obese adolescents.

Another critical area of focus is the relationship between obesity, metabolic syndrome, and cardiovascular health in children with chronic conditions such as chronic kidney disease (CKD). The study by Drozdz et al. provides evidence of the complex interplay between chronic kidney disease and cardiovascular risk, specifically left ventricular hypertrophy (LVH) in children. LVH, a major target organ damage in hypertension, is an important cardiovascular risk factor in CKD patients. This study shows that LVH in children with CKD was associated with multiple factors, among which the components of metabolic syndrome, hypertension, stage 5 chronic kidney disease, and growth deficit were the most significant. Understanding that metabolic syndrome, especially hypertension, are significant predictors of LVH in children with CKD suggests that managing these conditions is not only about treating kidney disease but also about mitigating cardiovascular risk.

Moreover, the study by Szczyrska et al. analyzes the effectiveness of structured weight-loss programs, showing significant health benefits from sustained participation, particularly among younger children. Participation in the full 12-month intervention within the ‘6-10-14 for Health’ program resulted in a greater long-term reduction in BMI and blood pressure when compared to non-participating children. Younger children, especially girls who participated in the intervention at age 6, benefited the most. These findings are pivotal, suggesting that the timing of interventions and adherence play crucial roles in determining long-term health outcomes.

The results of the study by Bomberg et al. are truly groundbreaking, as the results of previous publications on the efficacy of topiramate in the treatment of obesity in children and adolescents were based on clinical trials. These researchers analyzed the efficacy of combined therapy of topiramate plus lifestyle modifications in a real-life intervention. It was shown that in a sample of 282 children and adolescents with obesity, there was a significant percent BMI reduction at each time point (1.5-, 3-, 6-, and 12-month -1.2%, -1.9%, -3.2%, and -3.4%, respectively; all p<0.01), Nevertheless, no baseline characteristics evaluated were associated with response. This reflects a growing recognition of the need for a combination of therapeutic strategies to address the multifaceted challenges of pediatric obesity.

These articles together support a combined approach that includes endocrinological, cardiometabolic, pharmacological strategies, and lifestyle changes to tackle childhood obesity. This Research Topic not only enhances our understanding of the complex interactions between obesity and other health issues but also directs future research and clinical methods towards creating more targeted and effective treatments. By synthesizing these different views, this Research Topic makes an important contribution to discussions on child health. It stresses the importance of starting interventions early and customizing them to meet the specific needs of obese children. With such thorough methods, we aim to control and potentially reverse the growing problem of obesity-related health issues in the young (8).
